# Harvard Odontological Society

**Published:** 1891-05

**Authors:** 

**Affiliations:** Harvard Odontological Society


### HARVARD ODONTOLOGICAL SOCIETY.

The Harvard Odontological Society held its usual monthly meeting Thursday evening, October 23, 1890, at Young's Hotel, Boston.

The President, Dr. W. P. Cooke, in the chair.

Dr. E. C. Briggs read a paper on "Radical Operation for Removal of the Pulp."

(For Dr. Briggs's paper, see page 296.)

### DISCUSSION UPON DR. E. C. BRIGGS'S PAPER.

*Dr. Hamilton.*—Mr. President and Gentlemen,—In looking over my record-book I find that I have performed since last June nine operations of this nature. The first one was different from any that Dr. Briggs has mentioned; being a case where a large fungus growth nearly filled the cavity of a wisdom tooth. Little pain was caused by the insertion of the syringe-point, and the growth was completely paralyzed and removed; canals opened, cleaned, and filled. As I told the patient, it would be two years before I could fill the tooth permanently; the operation was a decided success.

Out of the nine cases, I have marked six that were complete successes. Of the successes, there were five fresh exposures, though there had been enough aching to show that it was useless to keep the pulps. One I had failed to devitalize with arsenious acid, and I managed to remove the nerve, but with a good deal of trouble and a good deal of pain. The exposure was distal on a lower molar, and the mouth was a very wet one. It was a pretty lively pulp, and the cocaine had very little effect on it. Another case had two old exposures, part of the pulp was sloughed and extremely sensitive. I managed to remove it, but complete success was not attained as in the other cases.

There is one thing to which I would call your attention,—if you cannot inject the cocaine thoroughly, you will not get relief from the pain.

In those cases where the destruction of the pulp was necessary, it was the custom before arsenic was discovered to put an instrument in and take out the nerve. Of course the pain was very intense, and the general idea to-day is that the removal of a pulp is accompanied by excruciating pain; the terror of that operation has lasted for forty years, ever since the use of arsenic. Now, here is the first improvement in destroying the pulp since the arsenious acid method was discovered,—the first real step to help us on in thirty or forty years, and I consider it the most important improvement since the rubber dam. We all know that arsenic is defective; nobody can tell what its effect is to be. We know it may ache, and probably will,—if it does not we are very grateful. The importance of this method is such that I trust all will try and see its advantage. Dr. Mason tried it the other day with complete success,—removing the pulp from an upper molar in which arsenic had failed; and I believe that the pain of putting in the hypodermic point, if it be done carefully, does not compare with the pain caused by the application of arsenious acid.

*Dr. Charles Briggs.*—I have had several cases, but I will describe but two. One was my own tooth, treated by Dr. Briggs, though not included in his paper. It was a lower molar, the pulp of which had been exposed for several years; it had not succumbed to arsenic, though it had been repeatedly exposed to its action. Dr. Briggs had capped it once, but I had the capping removed because the pulp got to be very rebellious. There seemed to be no way of destroying it except by the old method just mentioned, and I had concluded to allow it to take its course. My brother injected the cocaine, and the first pressure hurt considerably, but after waiting half a minute, or I think less, he took the engine instrument and went down into the pulp. I could not believe that he was doing so, as I felt absolutely nothing. He was called away and did not complete it at one operation. The next day he again proceeded, but the tooth was decidedly sensitive to the touch of an instrument. He treated same as before and removed the remainder of the pulp, filling the cavity without causing any pain. I have since experienced no discomfort from it whatever.

The second case was that of a hospital nurse who came to me with a wisdom tooth paining her. A dentist, in attempting to extract, had broken the tooth, leaving the pulp exposed and appearing above the surface of the root. She had suffered intensely for three days and nights, subsisting on a liquid diet, contact with solid food being agony. She could not endure any thermal changes. I took a piece of cotton saturated with a twenty-per-cent. solution of cocaine, touched it gently to the pulp, and waited a few seconds. I then took the hypodermic syringe, injected the cocaine, and removed the pulp in half an hour, the pain having all passed away.

*Dr. Taft.*—I should like to ask Dr. Briggs if applying the cocaine at one point of the pulp benumbs the whole, so that access can be had into any of the canals without causing the patient pain?

*Dr. Briggs.*—I apply it at the point of exposure, but once my tube is in, I force the cocaine down as hard as I can, so that I imagine it dissects its way all around the pulp. After the first pressure, which of course is a slight shock, I can then force it down steadily and hard, and discharge the contents of the syringe into the pulp-chamber. I then take a broach and remove the pulp.

*Dr. Clapp.*—Does Dr. Briggs prepare the cocaine fresh for every application?

*Dr. Briggs.*—I keep a small two-ounce bottle of the twenty-per-cent. solution at hand ready for use. I use it up rather freely, for, in taking up a whole syringeful that way, it doesn't take long to empty the vial.

*Dr. Clapp.*—Do you use an aqueous solution?

*Dr. Briggs.*—Yes.

*Dr. Potter.*—I would like to know one or two points in regard to this operation. In the first place, What shape of hypodermic point does Dr. Briggs use? The ordinary point is bevelled off decidedly, so that when you come to put in the point you do not insert the tube at all. It is a sort of entering wedge for the tube, so that this point would have to be buried in the pulp up to the entrance of the tube before any injection could be made.

The second question is, How does he guard against this surplus of cocaine being taken into the mouth? We all know that cocaine is a very powerful drug and needs to be carefully guarded against. I believe the medicinal dose is one-eighth to one-half a grain. In this operation evidently the cocaine is used quite freely. If the rubber dam were applied in each case, one could easily take care of the surplus, but in those cases where it is not used I should say it would be quite difficult to regulate that matter.

*Dr. Briggs.*—Those points are very good ones, and particularly the first one. I intended to have brought my hypodermic syringe with me,—or rather Dr. Hamilton's, for mine is used up. Unless you are very careful about them, they spoil; they must be washed out immediately. I spoke of using a hypodermic syringe, but that is not exactly what I do use; I file off that point, make it blunt, and some of the times that I have used it I have had fine gold tubes fastened into the end of the syringe. I intend to have a number of syringes made with the blunt end for this special purpose, both straight and curved, so that I can reach an exposure in any part of the mouth. That answers the first point. With regard to the second, concerning the surplus cocaine about the edges, of course, if the rubber dam is on, that answers the question; but where I cannot conveniently apply the rubber dam I wrap up very freely with thick, heavy doilies, which readily absorb the surplus. If it doesn't go down the throat, it doesn't matter much how strong it is, as it has no injurious effect in the mouth.

*Dr. Clapp.*—Have you ever observed any toxic effect from its use?

*Dr. Briggs.*—None at all. I have never heard of any toxic effect from cocaine except from those cases of injection into the mucous tissue. I do not think there has ever been a case from simple external application. I do not apprehend any trouble from its use by this method unless it is swallowed, and I am very careful about that, and have the patient wash out the mouth thoroughly before I begin to operate.

*Dr. Taft.*—Do you begin to operate immediately?

*Dr. Briggs.*—Immediately, because the effect passes off in a short time.

*Dr. Potter.*—I think this matter of guarding against the drug being swallowed is a very important one, and in connection with this operation attention ought to be called to it. It is very easy for a patient to swallow anything that is in the mouth while undergoing an operation. Cocaine is a very powerful drug, and if the rubber dam is not on it seems to me that with such a strong solution as twenty per cent., if the patient should swallow once, it might produce decided constitutional effects.

*Dr. Giblin.*—I would like to say a word in approval of the use of this drug. I suppose I average at least one pulpless tooth a day, and I have tried this drug in at least fifty cases since last spring. I have never used it with the hypodermic syringe, but I have been very happy with it in the form of crystals. I apply a few crystals of the drug in the cavity, which deliquesce in the pulp-chamber; when I find the pulp insensible, I remove it. I should think the use of the hypodermic syringe would be a decided improvement upon this method. There have been several cases in my vicinity showing toxic effects from the use of the drug injected in the mucous tissues or the subtissues. I have never used it in that way, but in the destruction of pulps I have had very favorable results from it.

*Dr. Taft.*—I should like to ask Dr. Briggs if he can tell us if the injection of chloroform or the bromide of ethyl into the pulp would not have the same effect, so that its removal could be accomplished without pain?

*Dr. Briggs.*—I cannot answer that question positively, as I have never tried either of them. I should not think they would. I have never succeeded in destroying the pulp by the use of cocaine crystals, as Dr. Giblin has, but it seems that in his hands it has been more successful. I have tried cocaine in various ways, but never until I used it in this form have I succeeded.

*Dr. Taft.*—I asked the question because Dr. Clifford has had very good results from the bromide of ethyl as a dentine obtundent, and, judging from its effect, it occurred to me it might be used with equally good results in removing a live and sensitive pulp.

*Dr. Clifford.*—I don't think it would succeed. The bromide of ethyl evaporates very quickly, and its obtunding action is from its rapid evaporation.

*Dr. Briggs.*—Those drugs have action, as a rule, as Dr. Clifford points out, rather from the freezing effect which is the result of their rapid evaporation. It does not last long, and is more of a freezing operation on the surface of dentine to which it is applied, but whether it would extend far enough to reach up into the roots, I doubt. Cocaine has no action whatever when used on the fibrils, but when applied in this way has a more penetrating and lasting influence than the other drugs.

*Dr. Taft.*—What is the effect of the bromide of ethyl on the nerves when producing anæsthesia? Is it to be understood there is a freezing of the nerves in such case?

*Dr. Briggs.*—It doesn't go into the tissue and touch each nerve separately; it is not so understood. It is not because the drug goes into the system and reaches every nerve and benumbs it, but it has its effect, as chloroform does, upon the nervous system generally.

*Dr. Werner.*—I attribute Dr. Briggs's success largely to the force he uses to inject the pulp-chamber. The force of the jet displaces the fluids there, exposing the clean surface of the pulp for the action of the cocaine. There seems to be some analogy between the effect of the impact of the syringe and the force used in driving the wooden peg into the pulp-chamber. I am acquainted with a man who practises this method of driving the wooden peg with apparently considerable satisfaction to himself, and evidently with the approval of his patients.

*Dr. Shepard.*—Dr. Briggs spoke of hemorrhage occurring during the operation; does he do anything to control it?

*Dr. Briggs.*—I have never made any application to control it; it stops in a few minutes; only it is remarkable that it should bleed so much after the application of the cocaine, especially in the case of a fresh exposure.

In regard to the points brought up by Dr. Werner, I am quite sure that the method does not have the terror which must certainly accompany the operation of driving a stick of wood into the pulp, and the pain really seems to be very slight. In the cases in which this method has been used the patients have all expressed themselves as being well satisfied, and I do not think that the first impact is any more than what would result if one had applied arsenic, and found it did not act, and must try it over again.

*Dr. Smith.*—I would like to know Dr. Briggs's explanation of the hemorrhage that takes place. If, after the injection, the cocaine anæsthetizes the pulp, why does it not control the hemorrhage as it does when used in operation for cataract?

While I am on my feet I wish to simply acknowledge my indebtedness for the great pleasure and instruction which I have derived from listening to this paper. While perhaps the use of cocaine in the different dental operations is not original, certainly the application of it as presented by Dr. Briggs is, and it seems to me important. Dr. Hamilton says that it is not in the discovery and multiplication of mechanical expedients that the surgeon of this day declares his superiority so much as in the skilful and judicious employment of those which are already invented; so the essayist seems to be the first to employ the application of cocaine to the pulp instead of arsenic, and it to me seems a very valuable addition to our knowledge of dentistry.

It has been stated by one gentleman that this operation was analogous to the driving of a stick of wood into the pulp. The essayist has not only conceived this idea, but he has put it in practice, and he has given us the evidence of the success of the method in a variety of conditions. In one case he put the point of the syringe into the pulp and told the patient to press the plunger into the syringe, which the patient did without causing himself pain. Now, if the gentleman thinks it is analogous to driving a stick of wood into the pulp, I would like to hear the result if some day he should apply the sharpened end of a piece of wood to an exposed pulp, and give the patient a mallet and ask him to drive the peg in.

Another gentleman has wisely spoken of the necessity for caution. Like all operations of dentistry where the use of dangerous drugs is necessary, it requires the skilled hand and good judgment. From the number of cases presented, and from the uniform success of the method, I think we have reason, as hearers of this essay, to put it in practice ourselves. I look upon it as valuable, and I feel personally indebted to the essayist for the instruction contained in his paper.

*Dr. Briggs.*—I cannot answer Dr. Smith's question. It possibly can be reasoned out by a little study. One's first idea would be that the pulp would be less liable to bleed than after almost any other operation, on account of the known fact that cocaine applied in other places restricts the hemorrhage. I have given the matter some thought, but I cannot explain it now. I have not been able to see into the reason why it should bleed so freely, but that it does I state as a clinical fact.

*Dr. Smith.*—My theory for its not controlling the hemorrhage is this: the capillary circulation in the pulp is entirely different from the capillary circulation in other membranes. Experience teaches us that the pulp is very slow to take up any medicament, and therefore really does not take up the cocaine into itself. The anæsthetic effect is produced on the surface, while in the mucous membranes the cocaine is taken into the capillary circulation and in that way tends to control the hemorrhage.

*Dr. Giblin.*—I have a theory concerning that also,—it may be wrong. In the use of arsenic it is advanced that it produces a suffocating effect at the apex of the canal, and in that way closes up the arterial circulation in the pulp. In the use of cocaine I believe that the pulp blood-vessels when cut in removing the pulp adhere to the walls of the cavity, and do not contract as a severed, torn artery in other places, and therefore bleed much longer. In extracting teeth we sometimes meet with a case where a tooth comes out very freely from the socket and is followed by arterial bleeding; and I have found that by inserting a long probe and tearing the connections of the artery, the hemorrhage would quickly subside, where in many cases styptics would not control.

*Dr. Clifford.*—Will Dr. Briggs tell us in what way it affects the hypodermic syringe?

*Dr. Briggs.*—The ordinary hypodermic syringe has a steel point which is simply coated over with gilding, and it rusts so rapidly I have great difficulty in getting it cleaned afterwards if it is left for a very short time without attention.

*Dr. Clifford.*—What would be the remedy?

*Dr. Briggs.*—Well, a gold point, but even then you would have to be very careful. It affects the whole syringe, injures the piston and the inner casing of the chamber.

*Dr. Clifford.*—It seems to act upon the syringe in about the same way as morphine?

*Dr. Briggs.*—Yes, only more so.

*Dr. Hamilton.*—In the upper jaw I prevent the escape of cocaine into the mouth; indeed, I fill most upper teeth by using a sheet of rubber dam as we commonly do a napkin, and then a napkin over it, thus preventing the latter getting damp. In cases where I had a large cavity, I would put over the points of my syringe one of the rubber cone that comes with Dunn's syringe, and by adjusting that carefully, putting the base of the cone towards the exposure, you can fit it so that the cocaine will not escape at all. By this method perhaps two or three drops will be all that you can force in, but I have succeeded in that way in two or three cases, and had no escape of cocaine into the mouth.

With regard to the action of bromide of ethyl or ether on the pulp, you can freeze to such an extent that there is no feeling. I have seen the record of it having been used in that way, but I think the operator in every case destroyed the nerves of more teeth than he meant to. The action cannot be confined to the diseased tooth, but the tendency is to freeze adjoining ones.

*Dr. Clifford.*—Did Dr. Hamilton inject the chloroform into the pulp, or did he spray it?

*Dr. Hamilton.*—It was used as a spray. I do not know that injection has ever been tried. I have not used it myself, I simply spoke of having seen the record of it.

Dr. Clapp, under specimens presented, showed a Pasteur filter from which all the water delivered passes through porcelain, and is claimed by Pasteur to be absolutely sterilized, bacteria being entirely eliminated. Its capacity at the pressure found in the basement of "Back Bay" houses is, for a single tube filter, about five gallons in twenty-four hours.

The filter is easily cleaned, and, so far as a few weeks' use can demonstrate, is entirely satisfactory, the water filtered is clear, and after standing for a month shows no deposit. [Professor Sedgwick, of the Massachusetts Institute of Technology, in his tests of this filter, found it to be as claimed above by Pasteur.—Ed.].

H. L. Upham, D.M.D.,
*Editor Harvard Odontological Society.*

